# Trigeminal, Visceral and Vestibular Inputs May Improve Cognitive Functions by Acting through the Locus Coeruleus and the Ascending Reticular Activating System: A New Hypothesis

**DOI:** 10.3389/fnana.2017.00130

**Published:** 2018-01-08

**Authors:** Vincenzo De Cicco, Maria P. Tramonti Fantozzi, Enrico Cataldo, Massimo Barresi, Luca Bruschini, Ugo Faraguna, Diego Manzoni

**Affiliations:** ^1^Laboratory of Sensorimotor Integration, Department of Translational Research and of New Surgical and Medical Technologies, University of Pisa, Pisa, Italy; ^2^Department of Physics, University of Pisa, Pisa, Italy; ^3^Institut des Maladie Neurodégénératives, University of Bordeaux, Bordeaux, France; ^4^Department of Surgical, Medical, Molecular Pathology and Critical Care Medicine, University of Pisa, Pisa, Italy; ^5^Department of Developmental Neuroscience, IRCCS Fondazione Stella Maris, Pisa, Italy

**Keywords:** ascending reticular activating system, locus coeruleus, pupil size, trigeminal nerve, visceral input, vestibular input, hemispheric imbalance, performance

## Abstract

It is known that sensory signals sustain the background discharge of the ascending reticular activating system (ARAS) which includes the noradrenergic locus coeruleus (LC) neurons and controls the level of attention and alertness. Moreover, LC neurons influence brain metabolic activity, gene expression and brain inflammatory processes. As a consequence of the sensory control of ARAS/LC, stimulation of a sensory channel may potential influence neuronal activity and trophic state all over the brain, supporting cognitive functions and exerting a neuroprotective action. On the other hand, an imbalance of the same input on the two sides may lead to an asymmetric hemispheric excitability, leading to an impairment in cognitive functions. Among the inputs that may drive LC neurons and ARAS, those arising from the trigeminal region, from visceral organs and, possibly, from the vestibular system seem to be particularly relevant in regulating their activity. The trigeminal, visceral and vestibular control of ARAS/LC activity may explain why these input signals: (1) affect sensorimotor and cognitive functions which are not directly related to their specific informational content; and (2) are effective in relieving the symptoms of some brain pathologies, thus prompting peripheral activation of these input systems as a complementary approach for the treatment of cognitive impairments and neurodegenerative disorders.

## Introduction: Functional Aspects of Brainstem Reticular Formation and Ascending Reticular Activating System (ARAS)

### Reticular Formation and ARAS

**Figure 1:**
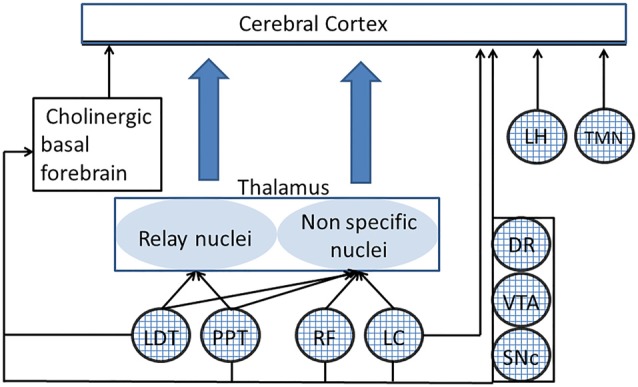
Connections of different ascending reticular activating system (ARAS) structures to the thalamo-cortical system. Only the principal connections of ARAS structures (indicated by textured circles) have been shown. PPT and LTD act on the specific thalamic relay nuclei and, together with the RF and the LC, on the diffuse thalamic system. DR, LC, VTA, lateral hypothalamus (LH) and tuberomammillary nucleus (TMN) have a direct access to the cerebral cortex. All ARAS structures connect to basal forebrain neurons, which are the source of cholinergic projections to the cerebral cortex. DR, dorsal raphe; LC, locus coeruleus; LDT, laterodorsal tegmental nucleus; LH, lateral hypothalamus; PPT, pedunculopontine nucleus; RF, reticular formation; SNc, substantia nigra pars compacta; TMN, tuberomammillary nucleus; VTA, ventral tegmental area.

The reticular formation (RF), a network of scattered cells that extends from the medulla to the hypothalamus and connects to most of brain structures, sustains nervous functions which are of crucial importance for body homeostasis and proper interaction with the environment, such as breathing, blood circulation (Guyenet, 2006, 2014), body (Luccarini et al., 1990; Pompeiano et al., 1991; Schepens and Drew, 2006; Takakusaki et al., 2016), head (Quessy and Freedman, 2004) and eye (Sparks et al., 2002) voluntary and reflex movements. Moreover, as discovered by the seminal work of Moruzzi and Magoun (1949) the RF controls arousal and sleep-waking cycle, shifting the pattern of thalamo-cortical activity between alternations of short periods of discharge and quiescence, which synchronizes within large populations (Steriade et al., 1993) and prolonged periods of continuous neuronal discharge, where only restricted populations of neurons nearby located (Steriade, 1995) or involved in the same task (Uhlhaas et al., 2009) show synchronized activity. The first pattern leads to an electroencephalographic (EEG) activity characterized by high amplitude and predominant low frequency components (synchronized or deactivated), typical of drowsiness and slow wave sleep, the second to a low amplitude, high frequency EEG (desynchronized or activated) pattern characteristic of wakefulness, but also present in the desynchronized sleep (Steriade et al., 1993; Steriade, 1995). Although there is evidence that the caudal region of the RF may include sleep-inducing, synchronizing structures (Magni et al., 1959), the studies of Moruzzi and Magoun showed the existence of an ascending reticular activating system (ARAS), which promotes the transition from a synchronized to a desynchronized EEG pattern, i.e., from sleep to wakefulness or from slow waves (synchronized) to rapid eye movement (REM, desynchronized) sleep (Steriade et al., 1993; Steriade, 1995). The ARAS extended up to the rostral region described by von Economo (1930) at the level of the junction between midbrain and thalamus, whose lesion leads to a persistent sleep in lethargic encephalitis. Later studies have shown that the ARAS includes not only glutamatergic RF neurons, as originally described, but also cholinergic and noradrenergic neurons within the dorsolateral pontine tegmentum (located in the pedunculopontine/laterodorsal tegmental (PPT/LTD) nuclei and the *Locus Coeruleus* (LC), respectively). Finally, also the histaminergic neurons in the tuberomammillary nucleus (TMN) of the posterior hypothalamic region, the peptidergic neurons in the lateral hypothalamus (LH) and the dopaminergic neurons in the mesencephalon and serotoninergic neurons in raphe nuclei (Jones, 2003; Saper et al., 2005) can be considered as ARAS components, although the precise action of the raphe neurons on cortical arousal has been questioned (Monti, 2011). These specific ARAS components regulate arousal through their connections with the specific thalamic relay nuclei and the diffuse thalamic system (Saper et al., 2005), the cerebral cortex (Saper et al., 2005) and the cholinergic basal forebrain neurons (Peyron et al., 1998; Jones, 2004; Fuller et al., 2011; Monti, 2011), which are the source of cholinergic input to the cortex, and play an essential role in the control of arousal (Fuller et al., 2011). The main connections of the ARAS with thalamo-cortical structures and basal forebrain are illustrated in Figure 1.

Among the different ARAS components, the LC has attracted a large amount of investigations, which, on top of elucidating the LC effects on neuronal activity and brain networks signaling, have also disclosed neurobiological LC actions that may have a deep impact on the brain dynamics, both in health and disease.

### The Noradrenergic LC System

It has been recently pointed out that the LC exerts a wide spectrum of influences on the brain, which span from tuning of neurophysiological activities related to sensorimotor/cognitive processes and setting of general brain excitability, to neurobiological functions such as the control of brain-blood barrier integrity, metabolic activity and neuroinflammatory processes (Mather and Harley, 2016). Some of these functions could place the LC at the core of the pathogenesis of neurodegenerative diseases (O’Mahony et al., 1994; Braak and Del Tredici, 2011; Del Tredici and Braak, 2013; Femminella et al., 2016).

The LC is located in the pontine brainstem, close to the forth ventricle. In humans, the LC consists of about 16,000 neurons for each side, which project virtually to the whole brain, with the exception of the basal ganglia, where LC fibers are limited to the nucleus accumbens (NAcb; see Berridge and Waterhouse, 2003 for reference) and to the substantia nigra pars reticulata (SNr; Vermeiren and De Deyn, 2017). However, due to these connections, the LC could play a role in the disorders of basal ganglia (Vermeiren and De Deyn, 2017). Interestingly, adult LC neurons tend to fire in synchrony, when their discharge rate is low, due to the presence of a weak electrotonic coupling between LC neurons (Heister et al., 2007). Such a synchronous firing could be reinforced by the existing electrical coupling of LC neurons to glial cells (Alvarez-Maubecin et al., 2000), which could in turn enhance the synchronous glutamate release from astrocytes (Pirttimaki et al., 2017).

### Neurophysiological LC Influences on the Brain

**Figure 2:**
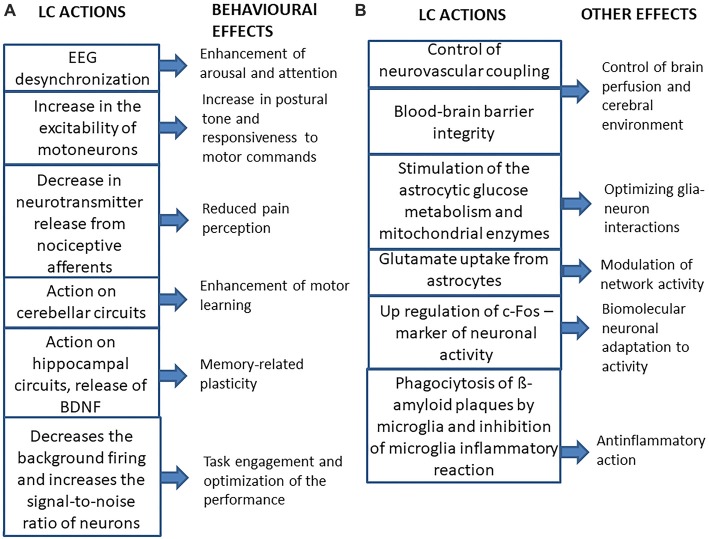
Summary of the main effects of LC activation on the brain and of their consequences. **(A)** Behavioral effects. **(B)** Other effects.

The main neurophysiological effects of LC on the brain and their behavioral consequences are summarized in Figure 2A. As expected for a structure which is a part of the ARAS (Jones, 2003; Lee and Dan, 2012), the LC desynchronizes EEG activity, enhancing arousal and attention (Berridge, 2008; Berridge et al., 2012), thus playing an important role in behavioral control (Aston-Jones et al., 1999; Aston-Jones and Cohen, 2005; Yu and Dayan, 2005; Berridge et al., 2012; Payzan-LeNestour et al., 2013). Moreover, its activity can also affect mood (Hirschfeld, 2000; see, however Liu et al., 2017).

The LC gives some contribution to the noradrenergic control of spinal motor activity, since it has been shown that activation of the LC, similarly to local norepinephrine (NE) application, increases the excitability of motoneurons (Fung et al., 1994), leading to a strengthening of postural tone and, possibly of the general responsiveness to motor commands. Moreover, LC stimulation has been shown to exert an inhibitory role on acute pain perception, by decreasing the release of neurotransmitters from nociceptive afferents (Llorca-Torralba et al., 2016). The LC noradrenergic neurons also control the gain of vestibulospinal reflexes, probably adapting them to the level of arousal (Pompeiano et al., 1991) and their projections to the cerebellar cortex seem to be necessary for the induction of those motor learning processes that modify vestibulo-ocular and vestibulospinal reflexes (van Neerven et al., 1991; Pompeiano et al., 1994). In this respect, noradrenergic projections to hippocampal structures play a fundamental role in memory-related plasticity phenomena (Sara, 2009, 2015; Sara and Bouret, 2012). Accordingly, NE stimulates the cerebral synthesis and release from astrocytes (Juric et al., 2006) of the brain-derived neurotrophic factor (BDNF), known to facilitate memory processes (Bekinschtein et al., 2014).

In the light of the multiple effects of LC on brain activity and plasticity, it is not surprising that there is a large body of experimental evidence indicating that the cognitive improvement elicited by physical exercise can be in part attributed to the activation of LC neurons by multiple reflex pathways of somatosensory and visceral origin (McMorris, 2016).

Microiontophoretic NE application on LC target neurons, and/or electrical or chemical (by local injection of neurotransmitters) LC activation in anesthetized animals, may enhance the responsiveness of cortical neurons to subthreshold stimuli, but also decrease the background firing of the cells more than the sensory-evoked response, thus leading to an increase in the signal-to-noise ratio of the neurons, which become responsive only to strongest inputs (Snow et al., 1999; Berridge and Waterhouse, 2003). This effect can be coupled to an increase in the responsiveness to both glutamate and GABA (Berridge and Waterhouse, 2003). The increase in signal-to-noise ratio of neuronal responses, is likely related to the suppressive role of the LC in the generation of epileptic discharges (Fornai et al., 2011) and takes place during the phasic activation of LC neurons. At variance, changes in LC background discharge affect neuronal responses to whatever input (Devilbiss and Waterhouse, 2011). In this respect, it has been recently proposed (Aston-Jones et al., 1999; Aston-Jones and Cohen, 2005) that phasic and tonic LC discharges underline two different behavioral states. The phasic discharge can be observed during waking, in response to task-relevant stimuli. The tonic discharge is very regular, ranges between 2 Hz and 15 Hz during active waking; it decreases to less than 2 Hz in quiet waking and drops further during slow wave sleep, being abolished during REM sleep. During waking, there are periods characterized by strong phasic excitation with a low level of tonic LC activity (phasic state), that correspond to a behavioral state called “exploitation”, which favors the engagement in specific tasks and optimizes performance. A high tonic discharge is associated with a reduced capability to generate burst firing (tonic state) and with a behavior called “exploration”, characterized by poor task performance, task disengagement and search of alternative behavioral choices. When the phasic discharge is present, the level of tonic LC activity is moderate. Transitions between phasic and tonic patterns change the responsiveness of LC targets: in the tonic mode there is a generalized increase in responsiveness, whereas in the phasic one, the increase in responsiveness is selective for the inputs related to the specific task in which the subject is engaged (Aston-Jones and Cohen, 2005).

### LC and Pupil Size

Interestingly, the LC activity can be indirectly estimated by measuring the pupil diameter (Silvetti et al., 2013; Hoffing and Seitz, 2015; Kihara et al., 2015; Einhäuser, 2016; Joshi et al., 2016; Reimer et al., 2016), since it has been shown, both in humans (Murphy et al., 2014) and in animals (Rajkowski et al., 1993, 1994) that pupil size is positively correlated to the LC activity. Pupil mydriasis is one of the most characteristic signs of arousal (Bradshaw, 1967; Bradley et al., 2008), it represents a fine indicator of brain state (McGinley et al., 2015) and, together with purposeful eyes motion, it has been taken has an indicator of persistent awakening status in the midpontine pretrigeminal preparation (Batini et al., 1959). The relation between noradrenergic LC neurons and pupil size is due to the control exerted by the LC on the preganglionic parasympathetic neurons of the Edinger-Westphal nucleus (Breen et al., 1983), which innervate the iris constrictor, inhibiting their discharge through an α2-mediated mechanism (Szabadi and Bradshaw, 1996; Samuels and Szabadi, 2008). This inhibition is necessary to increase the pupil size, since the tonic activity of the iris constrictor would prevent pupil enlargement by iris dilatator (Wilhelm et al., 2011).

### Other LC Influences on the Brain

Beyond its effects on neural activity, the LC regulates other neurobiological functions (see Figure 2B). LC axons are in intimate contact with the astrocytes (Paspalas and Papadopoulos, 1996; Cohen et al., 1997), where they enhance the intracellular Ca^2+^ inflow (Paukert et al., 2014), which has been shown to induce dilation of blood vessels (Koehler et al., 2009; Carmignoto and Gómez-Gonzalo, 2010; Petzold and Murthy, 2011; see also Girouard et al., 2010). In this respect, LC may increase brain perfusion (Toussay et al., 2013). Moreover, the LC is necessary for blood-brain barrier integrity (Harik and McGunigal, 1984; Kalinin et al., 2006), so it may affect the composition of the cerebral environment.

Moreover, noradrenergic activation increases glutamate uptake (Hertz et al., 2010), thus modulating network activity, stimulates the astrocytic glucose metabolism (Segal et al., 1980; Sorg and Magistretti, 1991) and the activity of mitochondrial enzymes (Hertz et al., 2010). It has been suggested that the LC optimizes the metabolic and functional interactions between neurons and astrocytes in the cerebral cortex and hippocampus, as well as in the nucleus basalis of Meynert: loss of LC neurons may disrupt these interactions leading to neuronal cell death in these areas (Hertz, 1989). In view of these LC effects is not surprising that an astrocytic dysfunction has been found in major depressive disorder (Chandley et al., 2013), where the noradrenergic transmission could be inadequate (Hirschfeld, 2000).

LC also controls gene expression as its stimulation induces ipsilateral c-Fos upregulation of cortical pyramidal cells and of a larger population of interneurons (Toussay et al., 2013), while its lesion abolishes the increase in c-Fos expression elicited by restraining (Stone et al., 1993) as well as by sleep deprivation (Cirelli et al., 1996). So, the LC controls the biomolecular cell adaptation to activity changes.

Finally, LC regulates neuroinflammatory processes, by inducing the phagocytosis of β-amyloid plaque by microglia (Heneka et al., 2002; Pugh et al., 2007; Kong et al., 2010), thus maintaining an adequate β-amyloid clearance (Kalinin et al., 2007) and by inhibiting the microglia inflammatory reaction (Yao et al., 2015). It has to be underlined that neuroinflammation seems to characterize also non pathological, but stressful conditions, such as sleep deprivation (Wadhwa et al., 2017).

Given the LC control of sensorimotor processing, learning and plasticity, metabolic processes, neurovascular coupling, inflammatory processes and gene transcription, a dysregulation of its activity may contribute to cognitive and arousal dysfunction and can be associated with a variety of behavioral and cognitive disorders (Berridge and Waterhouse, 2003; Aston-Jones and Cohen, 2005; Mather and Harley, 2016). Considering that LC degeneration is the early sign of both Alzheimer’s and Parkinson’s disease (Zarow et al., 2003; Grudzien et al., 2007; Mravec et al., 2014), is not surprising that LC dysfunction has been proposed as causal factor for such pathologies (Braak and Del Tredici, 2011; Del Tredici and Braak, 2013; see also O’Mahony et al., 1994; Femminella et al., 2016).

### ARAS Activity Regulation: Role of Sensory Afferents

According to the effects of ARAS activation on neural processing (Snow et al., 1999; Castro-Alamancos, 2002; Yu and Dayan, 2005; Devilbiss and Waterhouse, 2011; Dayan, 2012; McGinley et al., 2015), subtle changes in ARAS activity during the waking state may lead to changes in the sensory, motor and cognitive performance (Lee and Dan, 2012; McGinley et al., 2015). Although the sources of these changes in ARAS activity are largely undetermined (McGinley et al., 2015), the tonic discharge of ARAS’s neurons is sustained, beyond possible pacemaker properties (Chan and Chan, 1983; Sun et al., 1988), by the afferent inputs that these structures receive. Some of the afferent inputs to the ARAS are related to the control of specific somatomotor (Kuypers, 1981), oculomotor (Huerta and Harting, 1984) or vegetative (Guyenet, 2006; Ganchrow et al., 2014) functions associated with this network. Mutual connections between the different ARAS components are likely implicated in determining the level of arousal in relation to the sleep-wake cycle (Scammell et al., 2017). Other inputs arising from higher brain structures could allow to adapt the level of arousal on the basis of the cognitive (Leichnetz et al., 1984) and emotional state of the subject. In this respect, it has to be emphasized that all ARAS components strongly interact with the limbic system (Brodal, 1981; Veening et al., 1984; Ericson et al., 1991; Semba and Fibiger, 1992; Berridge and Waterhouse, 2003; Vertes and Linley, 2008; Lee et al., 2011). Finally, inputs from peripheral receptors may set the ARAS activation according to body/environmental conditions and sustain its tonic activity, as pointed out by the initial studies on RF (Starzl et al., 1951). The afferent control of ARAS by sensory afferents has three potential consequences:

1. Afferent discharge may regulate the excitability of brain networks involved in cognitive functions, which are not directly related to their specific sensory informational content, thus modulating cognition. In the same way, vestibular and neck signals acting on spinal projecting neurons belonging to the RF (Pompeiano et al., 1984) and to the LC (Manzoni et al., 1989) modulate the discharge of motor networks controlling the postural tone. So, based on this assumption, it could be expected that sensory afferent stimulation may boost cognitive performance by increasing the ARAS/LC discharge.
2. An asymmetry in the level of specific tonic sensory signals may lead to an asymmetric ARAS activity and, in turn, to an imbalance in hemispheric excitability. There is indeed evidence that a lesion-induced hemispheric imbalance may lead to specific cognitive deficits which are abolished by a second symmetric lesion (Lomber and Payne, 1996). So, asymmetries in the level of sensory afferent inputs may potentially induce cognitive dysfunctions, which could be prevented by counteracting the afferent asymmetry. Alternatively, an asymmetric stimulation of specific sensory afferents may compensate for hemispheric imbalance (Rubens, 1985; Bottini et al., 1995; Schiff and Pulver, 1999).
3. Finally, due to the neurobiological influences that LC exerts on the brain, a disruption of sensory afferent discharge, impinging on the LC itself, may potentially influence the development of neuroinflammatory and degenerative processes in brain structures.

In the next three sections, we will review a large body of evidence supporting points 1–3, arising from experiments investigating the influences exerted on the brain by modifications of the afferent trigeminal, visceral and vestibular inputs.

## Trigeminal, Visceral and Vestibular Influences on Brain Functions Through ARAS and LC

### Acute Trigeminal Influences: Effects of Afferent Stimulation

Previous studies report that chewing improves cognitive processing speed (Hirano et al., 2013), alertness (Allen and Smith, 2012; Johnson et al., 2012), attention (Tucha et al., 2004) and intelligence (Smith, 2009). Objective modifications can be observed in the reaction times (shortening; Tucha et al., 2004; Allen and Smith, 2012; Hirano et al., 2013), in the event-related potentials latencies (decreasing, see Sakamoto et al., 2009, 2015) and in the cerebral blood oxygen-dependent signal (increasing, see Hirano et al., 2013). Interestingly, the effects of chewing were not replicated by rhythmic finger motor activity or by rhythmic jaw movement in the absence of chewed material (Sakamoto et al., 2015).

So, orofacial input is particularly effective in enhancing arousal, thus boosting performance. In fact, 2 min of chewing a hard pellet may boost performance and task-induced mydriasis, while a bilateral handgrip exercises of the same duration do not (Tramonti Fantozzi et al., 2017). These data are consistent with the observation that, in the *encephale isolé* preparations, where ARAS and LC have been disconnected from the spinal cord, an additional lesion of the trigeminal, but not of the other cranial nerves afferents, triggers the transition of the desynchronized EEG activity towards a synchronized sleeping pattern (Roger et al., 1956).

However, the positive effects of chewing on cognitive performance are not observed following sleep deprivation, although, in this condition, chewing may again improve alertness and mood (Kohler et al., 2006). This result is not surprising, since sleep deprivation enhances LC activity, as documented by the c-Fos expression (Pompeiano et al., 1992). A higher LC background discharge would prevent the trigeminal-induced enhancement in phasic LC activity, which seems necessary for focusing the attention on the task, and for enhancing performance (Aston-Jones et al., 1999; Aston-Jones and Cohen, 2005).

### Acute Trigeminal Influences: Effects of Balancing and Unbalancing Occlusion

Recent experiments have shown that the presence of a sensorimotor trigeminal imbalance (related to occlusal problems)—consisting in an asymmetric activation of masseter muscles during clenching—is strongly correlated to an asymmetry in pupils size, either in the normal jaw resting position, with the arches few millimetres apart, or with the teeth in contact (De Cicco et al., 2014, 2016). Since pupil size is considered as a reliable indicator of LC activity and its asymmetry is strongly reduced following removal of the trigeminal imbalance by occlusal correction, these data suggest that the trigeminal imbalance makes the LC discharge asymmetric. Removal of the asymmetries in sensorimotor trigeminal activity, not only makes pupils symmetric, but also enhances the performance in a cognitive task and the mydriasis associated with an haptic task (De Cicco et al., 2014, 2016), thus suggesting that trigeminal-induced imbalance in LC activity (and, as a consequence in hemispheric excitability) is detrimental to the performance and to cortical arousal, whose level is indicated by task induced mydriasis (Bradshaw, 1967; Bradley et al., 2008). The effects of hemispheric imbalance on behavior are documented by the observation that unilateral brain lesions lead to severe cognitive deficits (Lomber and Payne, 1996; Kerkhoff, 2001) which can be greatly reduced by a second, symmetric lesion on the opposite side (Lomber and Payne, 1996), or by asymmetric sensory stimulation (Kerkhoff, 2001). Consistently, manipulation of the trigeminal information may either induce or relieve asymmetries in brain excitability, thus modifying the cognitive performance (De Cicco et al., 2014, 2016).

### Trigeminal Pathways to ARAS and LC

**Figure 3:**
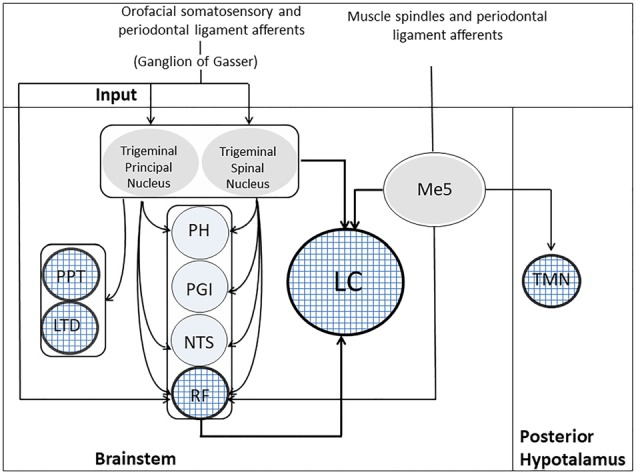
Trigeminal Pathways to ARAS’s structures. Muscle spindles and periodontal ligament receptors, through the Me5, project to five ARAS’s structures, indicated by the textured circles: LC, RF, PPT, LDT and TMN. Orofacial somatosensory/periodontal ligament afferents reach the trigeminal principal/spinal nuclei and the RF through the ganglion of Gasser. The trigeminal principal/spinal nuclei project to both LC and RF, as well as to PPT and LTD. Indirect pathways from trigeminal nuclei to the LC may run through the PH, the PGI, the NTS and the RF. LC, Locus Coeruleus; LTD, laterodorsalis tegmental nucleus; Me5, mesencephalic trigeminal nucleus; NTS, nucleus of the tractus solitarius; PGI, nucleus paragigantocellularis; PH, prepositus hypoglossi; PPT, pedunculopontine nucleus; RF, reticular formation; TMN, tuberomammilary nucleus.

There are several pathways that may bring trigeminal input to the ARAS and the LC: the most important are shown in Figure 3. First of all, primary sensory fibers, including proprioceptive jaw muscle spindles from mesencephalic trigeminal sensory nucleus (Me5) reach directly the RF (Brodal, 1981; Dessem et al., 1997). Me5 afferents transporting proprioceptive information from periodontal ligaments and muscle spindles of the oral cavity project also to hypothalamic TMN neurons (Fujise et al., 1998; Sakata et al., 2003). Secondary fibers from trigeminal nuclei reach the RF (Brodal, 1981; Shammah-Lagnado et al., 1987, 1992; Schmid et al., 2003) and, in addition, the diffuse thalamic system (Krout et al., 2002). All trigeminal nuclei, including the Me5, project to the LC (Cedarbaum and Aghajanian, 1978; Luo et al., 1991; Craig, 1992; Couto et al., 2006; Dauvergne et al., 2008). In particular, it has been claimed, on the basis of fluorogold transport from Me5 cells to neurons within the boundaries of the LC (Fujita et al., 2012), that these two structures are electrotonically coupled (Matsuo et al., 2015). Moreover, trigeminal signals may reach the LC also through the nucleus of tractus solitarius (NTS) and the RF (Zerari-Mailly et al., 2005; Schwarz and Luo, 2015).

All the fibers mentioned above terminate outside the core of the nucleus, in a region where only LC dendrites are located. So they are probably less effective in driving LC neurons than those impinging upon the cell bodies (Aston-Jones et al., 1991). Inputs to dendrites, however, could affect the electrical coupling of LC neurons, which occurs at dendritic level (Ishimatsu and Williams, 1996). The core of the LC, where cell bodies are located, receive inputs, which arises from the hypothalamic orexinergic neurons (Peyron et al., 1998), the nucleus paragigantocellularis (PGI) and the nucleus prepositus hypoglossus (PH; Aston-Jones et al., 1986), PGI sending excitatory (glutamatergic) and PH inhibitory (GABAergic) fibers (Aston-Jones et al., 1991). Both PGI and PH receive afferents from the trigeminal nuclei (Lovick, 1986; Buisseret-Delmas et al., 1999). So, through these indirect pathways the orofacial input is likely to exert its strongest influence on LC activity. The effectiveness of trigeminal input in LC activation is documented by the increase in c-Fos expression induced at this level by trigeminal stimulation (Mercante et al., 2017). Finally, the principal and the spinal trigeminal nuclei project to cholinergic PPT and LDT neurons (Semba and Fibiger, 1992).

### Long-Term Effects of Trigeminal Afferents

There is evidence that, in addition to these short-term effects on performance and task-induced mydriasis, trigeminal signals may lead to long-term effects on the central nervous system (CNS), that may be helpful in preventing degradation of brain functions (see Ono et al., 2010 for reference). In particular, epidemiological studies have verified that tooth loss before 35 years of age or unilateral masticatory activity represents a significant risk factor for developing dementia or Alzheimer’s disease (AD; Weijenberg et al., 2011; Okamoto et al., 2015), while masticatory rehabilitation allowed aged animals to recover spatial abilities (Mendes Fde et al., 2013). Moreover, it has been documented, in aged animals, that bilateral molar extractions, leading to long-term masticatory dysfunction, decreases the number of pyramidal cells in the hippocampal CA1 and gyrus dentatus (Oue et al., 2013), with impairments in spatial learning and memory in water maze tests (Kato et al., 1997; Onozuka et al., 1999). These deficits seem to increase with aging and time after tooth loss (Onozuka et al., 2000) and can be replicated by soft-diet feeding (Tsutsui et al., 2007). Teeth loss also increases, at hippocampal level, the proliferation and the hypertrophy of the astrocytes, as it occurs following neuronal degeneration and senescence processes (see Onozuka et al., 2000), while decreasing c-Fos expression during spatial task (Watanabe et al., 2002), dendritic spines density (Kubo et al., 2005) and neurogenesis (Aoki et al., 2010). It is worth of note that reduction in number of c-Fos-positive cells in the hippocampal CA1 region and spatial learning impairment were partially antagonized by restoring the lost molars with artificial crowns (see Watanabe et al., 2002). In addition to teeth loss, also a soft diet may depress spatial learning in aged animal (Frota de Almeida et al., 2012), leading to changes in size and laminar distribution of astrocytes (Frota de Almeida et al., 2012) and reducing the BDNF levels (Yamamoto et al., 2008) and hippocampal neurogenesis (Yamamoto et al., 2009).

Finally, malocclusion leads to increase of apoptosis markers in the hippocampus and to accumulation of β-amyloid (Ekuni et al., 2011), which is one of the clinical symptoms associated with AD (Sisodia et al., 1990). Some authors point to stress-induced malocclusion with elevation of glucocorticoid levels (Yoshihara et al., 2009) as a causal factor for the expression of this constellation of symptoms which remind brain neurodegenerative processes. This hormonal response is abolished following destruction of the ventral ascending LC projections (Yoshihara and Yawaka, 2001), thus indicating that LC may link a disrupted trigeminal input to a glucocorticoid induced trophic dysfunction. However, a trophic dysfunction may also arise from a molarless condition-impaired LC discharge, leading to accumulation of β-amyloid, with the development of local inflammation and neurodegenerative processes (Ekuni et al., 2011), to a drop in local BDNF levels (Juric et al., 2006), to altered neuron-astrocyte interaction with metabolic dysfunctions at neuronal level (Hertz, 1989), to blood brain barrier dysfunction (Harik and McGunigal, 1984) and, finally to altered neurovascular coupling (Koehler et al., 2009; Carmignoto and Gómez-Gonzalo, 2010; Petzold and Murthy, 2011). All these factors may contribute to the degenerative processes that seem associated with impairment of the trigeminal functions.

In the light of all this body of experimental evidence, it appears that the lack of masticatory stimulation could be a major problem in the elders, where general motor activity, which may represent another way to drive LC discharge (McMorris, 2016), is generally reduced. In order to face the consequence of a deteriorated trigeminal input in the older age, strategies of “environmental enrichment” could be attempted, which improve cognition during aging (Stuart et al., 2017) and are likely to boost the LC activity (Saari et al., 1990).

### Trophic Trigeminal Influences on LC

How can a disruption of trigeminal afferent input impair LC activity? Beyond the consequences of a lack of excitation on the side where the trigeminal input is decreased, trigeminal fibers may also exert a trophic action on LC neurons by carrying neurotrophic factors from the periphery. Normally, the masseter muscle and its neuro-muscular spindles synthetize and release BDNF and neurotrophin-3/glial cell line-derived neurotrophic factor (GDNF), respectively, which prevent cell body atrophy of proprioceptive Me5 (Fan et al., 2000), of LC and autonomic neurons (Arenas and Persson, 1994; Buj-Bello et al., 1995), as well as of astrocytes (Henderson et al., 1994) and regulate LC neurons differentiation (Traver et al., 2006). Thus, a reduction of trigeminal proprioceptive signals may lead to reduced levels of neurotrophic factors for LC neurons, favoring the development of their dysfunction. Such a dysfunction may also extend to the glia, due to the large electrical coupling between Me5-LC cells and local astrocytes (Alvarez-Maubecin et al., 2000; Moore and O’Brien, 2015) and to the spread of local injury to the adjacent coupled elements, via gap junctions (bystander killing, see Moore and O’Brien, 2015).

### Effects of Non-trigeminal Orofacial and Visceral Inputs

It is known that some somatosensory orofacial inputs from the outer ear reach the brain through the vagus nerves (Brodal, 1981). Moreover, visceral afferents from the pharynx and the larynx travel in the glossopharyngeal and vagal nerves. These orofacial, non-trigeminal, pharyngeal and laryngeal fibers reach the trigeminal nuclei and the NTS (Brodal, 1981; Altschuler et al., 1989; Grélot et al., 1989; Chien et al., 1996), the latter receiving a wide spectrum of visceral afferents from the inner organs. It is likely that all this information may play a role similar to that of trigeminal orofacial afferents in modulating ARAS/LC excitability and cognitive functions.

Recent investigation has shown, in fact, that vagal stimulation in humans may affect mood, by decreasing depression symptoms (Milby et al., 2008), while subdiaphragmatic vagal section in animals leads to differential changes in the level of innate and learned anxiety (Klarer et al., 2014).

It is also known that and altered gut-brain axis feedback may contribute to mood disorders (Lerner et al., 2017). Stimulation of vagal afferents also improves memory consolidation in humans (Vonck et al., 2014) and animals (Clark et al., 1998), as well as hippocampal long-term potentiation (Zuo et al., 2007); moreover, it enhances the velocity of action selection during execution of sequential operations (Steenbergen et al., 2015). Interestingly, an improvement in memory has been observed also in subjects affected by AD (Vonck et al., 2014). On the other hand, vagal stimulation in humans seems to be detrimental for cognitive flexibility (Ghacibeh et al., 2006), while, in animals, vagotomy increases the ability in responding to instruction reversal (Klarer et al., 2017). Although several central visceral pathways could be involved in these vagal effects, most of the latter seems to be consistent with a vagal activation of the noradrenergic system: in fact an increase in NE release, due to LC activation, would improve depression symptoms (Hirschfeld, 2000; see, however Liu et al., 2017) boost memory and LTP (Hansen, 2017), facilitate sequential operations (Mückschel et al., 2017), while decreasing cognitive flexibility (Beversdorf et al., 2002). The effect on mood could be in part related to an enhanced release of melatonin from the pineal gland (Zarate and Manji, 2008), elicited by LC activation (Mitchell and Weinshenker, 2010).

Vagal stimulation directly increases the activity of LC neurons (Dorr and Debonnel, 2006; Manta et al., 2009) and indirectly—via the LC (Manta et al., 2009)—also that of raphe neurons (Dorr and Debonnel, 2006). Moreover, vagal stimulation leads to an increase of c-Fos expression within the LC (Gieroba and Blessing, 1994). It is not surprising, therefore, that vagal stimulation increases the pupil diameter (Desbeaumes Jodoin et al., 2015) and enhances NE release at the level of the CNS (Hassert et al., 2004; Roosevelt et al., 2006; Follesa et al., 2007; Raedt et al., 2011). Similarly to the trigeminal system, also the viscero-sensitive system could be involved in neurodegenerative processes. The NTS, in fact, is particularly sensitive to the effects of circulating inflammatory mediators (Hermann et al., 2001; Daulatzai, 2012) and it is known that prolonged activation of the peripheral immune system may promote microglia activation and neuroinflammation within the CNS (Godbout et al., 2005; Henry et al., 2009). So, similarly to teeth removal, peripheral inflammatory processes may lead to disruption of an important pathway impinging on the LC.

On the other hand, vagal stimulation exerts important trophic effects on the brain: it has been documented, in fact, that activation of vagal afferents induces an increased production of BDNF and NGF (Follesa et al., 2007) within the brain, together with an enhancement of hippocampal neurogenesis (Revesz et al., 2008); moreover, vagal stimulation also decreases neuroinflammatory responses (Meneses et al., 2016). These actions, possibly related to LC activation (Coradazzi et al., 2016; Mello-Carpes et al., 2016), may contribute to the positive effects of vagal stimulation in AD.

### Visceral Input to ARAS/LC

**Figure 4:**
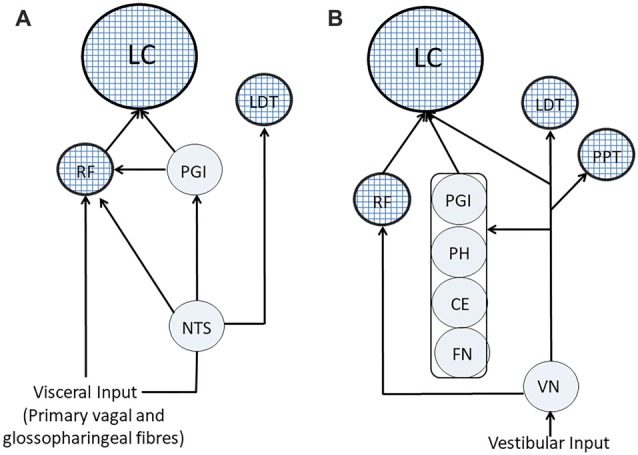
Visceral and vestibular pathways to ARAS’s structures. **(A)** Visceral pathways. **(B)** Vestibular pathways. ARAS structures are indicated by textured circles. CE, cerebellar cortex; FN, fastigial nucleus; LC, Locus Coeruleus; LTD, nucleus laterodorsalis tegmenti pontis; NTS, nucleus of the tractus solitarius; PGI, nucleus paragigantocellularis; PH, prepositus hypoglossi; PPT, pedunculopontine nucleus; RF, reticular formation; VN, vestibular nuclei.

Visceral input has a wide access to ARAS structures: in fact, as shown in Figure 4A), it may reach directly the RF through primary vagal and glossopharyngeal fibers, as well as from NTS (Brodal, 1981; Ganchrow et al., 2014). Moreover, the NTS projects to the LC (Cedarbaum and Aghajanian, 1978; Van Bockstaele et al., 1999; Ruffoli et al., 2011), which may also receive the visceral input through the nucleus paragigantocellularis, targeting neuronal cell bodies (Lovick, 1986). Finally, the NTS projects to the LDT (Cornwall et al., 1990), thus influencing also the discharge of cholinergic neurons.

### Vestibular Influences: Balancing the Brain

Recent evidence indicates that vestibular information could play a role in neural processing, beyond the contribution to a multisensory representation of the self and of the space (Britten, 2008; Zu Eulenburg et al., 2013; Hitier et al., 2014; Pfeiffer et al., 2014; Yoder and Taube, 2014). In fact, asymmetric labyrinthine activation ameliorates the cognitive deficits induced by lesions of the right parietal cortex (Rubens, 1985; Bottini et al., 1995; Schiff and Pulver, 1999) and of other brain regions (Schiff and Pulver, 1999), in humans. Attenuation of the symptoms was observed by either increasing the afferent vestibular discharge on the lesioned side, or by decreasing it on the contralateral side. Patients with right cortical damage are unconscious of body parts (asomatognosia) contralateral to the lesion and present spatial (hemi) neglect, i.e., failure to attend and respond to sensory stimuli contralateral to the lesion side (Kerkhoff, 2001). These alterations are not only due to the nervous tissue damage, but also to the imbalance in the activity of right and left hemispheres. It has been observed, in fact, that hemi neglect due to parietal lesions, in animals, ameliorate following a second, symmetric lesion produced in the contralateral hemisphere (Lomber and Payne, 1996). In other words, two symmetric lesions are less impairing than a unilateral one. A possible interpretation of these effects of vestibular stimulation is that these afferents exert a tonic control on the excitability of brain structures and that appropriate modulation of their discharge can decrease the inter-hemispheric imbalance, thus attenuating its neurological consequences.

### Vestibular Input: Cognitive Effects

In normal subjects, labyrinthine stimulation may affect several cognitive functions somehow related to self and space representation, such us the localization of tactile stimuli on the hand (Ferrè et al., 2013c), the perceived size of the touched objects (Lopez et al., 2012), the perceptual somatosensory illusion of a “fake” rubber hand ownership (Lopez et al., 2010) and line bisection task (Ferrè et al., 2013b).

Moreover, vestibular deficits impair the metric properties of space representation, as inferred by mental imagery tests (Péruch et al., 2011) and are associated with personality changes, further suggesting that vestibular sensation is implicated in the sense of self. These are depersonalization and derealization symptoms such as feeling “spaced out”, “body feeling strange” and “not feeling in control of self” (Smith and Darlington, 2013). It has to be acknowledged that neural processing related to self and space representation benefit from the informational content of vestibular input and may be directly affected by its manipulation. So, the evidence that vestibular signals exert a tonic modulatory action on cognitive functions is not so compelling as for trigeminal and visceral information.

However, vestibular signals may also affect cognitive performances not directly related to self and spatial perception. Labyrinthine stimulation has been shown to ameliorate tactile allodynia, which is attributed to a hemispheric imbalance (McGeoch et al., 2009), and to improve face perception deficits in post-stroke subjects (Wilkinson et al., 2005). Moreover, it influences also the balance between novel and routine motor responses to acoustic and visual stimuli (Ferrè et al., 2013a) and speed of visual memory processing (Wilkinson et al., 2008).

### Vestibular Pathways to ARAS and LC

At least in part, the effects of vestibular afferents on cognitive functions could be mediated by the central noradrenergic system arising from the LC, whose neurons are affected by the labyrinthine input (Manzoni et al., 1989; Kaufman et al., 1992), as confirmed by the observation that vestibular stimulation induces changes in pupil size (Kitajima et al., 2010, 2013), which is a reliable indicator of LC activity (Rajkowski et al., 1993, 1994; Murphy et al., 2014). It should be reminded that the vestibular influences on the LC have been proposed as the cause of the development of hemispheric specialization, since, during foetal development, the asymmetric position of the head is likely to favor activation of the left labyrinth during maternal locomotion, leading to an asymmetric development of several central structures (Previc, 1991).

As shown in Figure 4B, fibers arising from the spinal vestibular nucleus may directly reach the LC (Schwarz and Luo, 2015), but vestibular information can also travel along indirect pathways running through LC-projecting structures (Schwarz and Luo, 2015), such as the cerebellar cortex and the fastigial nucleus (Brodal, 1981), the PH (Belknap and McCrea, 1988) and the nucleus paragigantocellularis (Lovick, 1986). Finally, vestibular nuclei project to the RF (Brodal, 1981; Lai et al., 1999; Matesz et al., 2002) and to PPT and LDT cholinergic neurons (Semba and Fibiger, 1992; Aravamuthan and Angelaki, 2012).

## Translational Exploitation of Trigeminal, Visceral and Vestibular Control of ARAS/LC Activity

The impact of trigeminal, visceral and vestibular signals on ARAS and LC may lead to interesting clinical applications. Infraorbital trigeminal branch stimulation (ITS; DeGiorgio et al., 2003, 2006) has been utilized in humans for seizure control in epileptic patients refractory to antiepileptic drug treatment. This effect depends upon the activation of LC noradrenergic system (Zare et al., 2014), whose anti-seizure effect has been described in details (Weinshenker and Szot, 2002). Similar results have been also obtained with stimulation of afferent vagal fibers (Groves and Brown, 2005), which also activate LC and raphe system through the NTS (Krahl and Clark, 2012).

Recently, the observation that ITS induces an improvement of the mood, promoted the use of the trigeminal nerve stimulation (TNS) in subjects with major depressive disorder (Cook et al., 2013), with a significant improvement of the patient’s symptoms. This positive effect was attributed to the activation of the trigeminal nerve projections to LC and raphe nuclei (Cook et al., 2013), leading to an increase of serotonin and NE within the brain.

ITS was also tested in adult patients affected by fibromyalgia, a pain syndrome associated with neurological deficits in intracortical modulation as well as in young people affected by attention-deficit/hyperactivity disorder, a condition characterized by abnormal levels of inattention and/or hyperactivity/impulsivity. In the former case, TNS can improve pain and depressive symptoms (Shiozawa et al., 2014), while in the second it ameliorates attention, mood and sleep quality (McGough et al., 2015). Yet, the observed results could be related to trigeminal activation of LC/raphe nuclei, possibly driving the antinociceptive control system (Stamford, 1995) and normalizing monoamine levels in the brain. Of course, all these investigations could be extended to electrical activation of the vestibular nerve.

Another interesting field of application for peripheral activation of signals with a high impact on ARAS structures could be that of neurodegenerative disease. A recent approach to the treatment of AD has been to modulate brain activity through electrical stimulation of peripheral nerve and deep brain structures (see Laxton et al., 2014, for reference). As recently proposed, when neurons die synaptic activity on target cells is reduced, leading to upregulation of AMPA receptors, in an attempt to stabilize the global activity of the circuit (Palop and Mucke, 2010) and, as a consequence, to an increased cell excitability (Fröhlich et al., 2008). Such an increase will be coupled with a dysregulation of Ca^2+^ homeostasis due to β-amyloid (Demuro et al., 2010), leading to a greater influx of Ca^2+^ into the cell and making cell death (apoptosis) more likely. Consistently, neurons are more excitable in the brain of AD patients (Busche et al., 2008) resulting in an increased frequency of seizures. Based on these findings, it has been proposed that enhancing the circuit activity by electrical stimulation of peripheral or central structures may counteract the hypoactivity of the circuit, thus avoiding the reactive increase in excitability and the spreading of neurodegeneration (Rowan et al., 2014), assumption validated by neural network studies (Rowan et al., 2014). According to this hypothesis, long-term deep (Smith et al., 2012) or transcranial electrical (Hansen, 2012) or magnetic brain stimulation (Rabey et al., 2013) seem to improve the cognitive performance of patients, and similar results can be obtained by vagal nerve stimulation (Laxton et al., 2014). As compared to direct brain stimulation techniques, peripheral nerve stimulation is technically easier; moreover, it may activate the same neural circuits engaged in physical and cognitive training (activities which can be of help in counteracting AD), but without being limited by patient’s cooperation and motivation.

Since the LC is implicated in AD initial stages, stimulation of trigeminal proprioceptive afferents, which could be electrically coupled to LC neurons (Fujita et al., 2012; see however Tramonti Fantozzi et al., 2017), could rescue their activity and trophic condition. Indeed, chewing helps to maintain cognitive functions while masticator deficiency is associated with development of dementia (Teixeira et al., 2014). Moreover, several studies demonstrate that the loss of the molar teeth (molarless condition) may induce hippocampal senescence, characterized by a reduction of CA1 subfield neurons and by proliferation and hypertrophy of glial fibrillary acidic protein-labeled astrocytes (GFAP) in the same subfield (Onozuka et al., 1999, 2000). Also, Parkinson’s disease patients could benefit from trigeminal stimulation, since LC degeneration also occurs in this pathology at early stages.

Another interesting field of application of trigeminal stimulation could be the treatment of functional impairments induced by hemispheric asymmetries. It is known that asymmetries in trigeminal (as well as vestibular) signals induced by malocclusion are detrimental for performance and their elimination by occlusal correction improves cognitive performance in patients affected by AD (De Cicco, 2012), as well as in normal subjects (De Cicco et al., 2014, 2016). An asymmetric brain activity may also arise from unilateral lesions and, in this instance, activation of vestibular afferents on the appropriate side brings to a relief of patient’s symptoms (Rubens, 1985; Bottini et al., 1995; Schiff and Pulver, 1999). This effect is likely dependent upon the vestibular connections with ARAS and LC, which allow to rebalance hemispheric activity, thus prompting the use of trigeminal stimulation for the same purpose, lacking secondary undesired effects, such as vertigo. Finally, brain asymmetries may also result from environmental factors, such as exposure to ionizing radiations, shown to be associated with a higher incidence of neuropsychiatric disorders (Loganovsky et al., 2008). This is particularly relevant for subjects working in cardiac catheterization units, who are exposed to a high cumulative levels of low-doses of ionizing radiations, specifically at the left hemisphere, given the standard setup in the catheterization lab (Andreassi et al., 2016). It has been shown that this condition is associated with impairment of cognitive performance and may therefore represent a professional risk (Marazziti et al., 2015). Since irradiation affects brain activity (Loganovsky and Yuryev, 2001; Loganovsky and Kuts, 2016), it is likely that an asymmetric exposure to ionizing radiations leads to an asymmetric brain activity and, as a consequence to cognitive impairments. Even in this instance TNS, unilaterally applied could be used to reduce asymmetries in brain excitability, thus resulting in an improvement of cognitive performance.

## Author Contributions

VC and MPTF planned the manuscript layout, performed bibliographical research and wrote part of the manuscript. EC performed bibliographical research and wrote part of the manuscript. MB and LB performed bibliographical research and provided critical insights. UF and DM conceived and wrote the manuscript.

## Conflict of Interest Statement

The authors declare that the research was conducted in the absence of any commercial or financial relationships that could be construed as a potential conflict of interest.
